# Supplementary material for: Heterogeneity-induced lane and band formation in self-driven particle systems
Source: Sci Rep. 2022 Mar 19;12:4768. doi: 10.1038/s41598-022-08649-4 (PMC8934355; doi:10.1038/s41598-022-08649-4)
Supplement: Supplementary file 1 — Supplementary Information. [file 41598_2022_8649_MOESM1_ESM.pdf]

Heterogeneity-induced lane and band formation in self-driven particle systems

Supplementary Materials

Basma Khelfa[1], **Raphael Korbmacher**[1], **Andreas Schadschneider**[2], **and Antoine Tordeux**[1,*]

[1]School for Mechanical Engineering and Safety Engineering, University of Wuppertal, Wuppertal, Germany
[2]Institute for Theoretical Physics, University of Cologne, Cologne, Germany
[*]e-mail: tordeux@uni-wuppertal.de

We use the collision-free (CF) model in the manuscript to analyse by simulation the two heterogeneity models M_1 Eq. (1) and M_2 Eq. (2). In these supplementary materials, we present identical simulation experiments as those carried out in the manuscript by using the Social Force (SF) model[1]. Generally speaking, similar phase transitions to lane and band patterns occur when using, respectively, the static heterogeneity models M_1 or the dynamic model M_2. In the following, we first present the simulation results before providing technical details on the social force model.

Simulation results We simulate using the SF model the evolution of 45 agents on a 9×5 m rectangle with periodic boundaries (see Sec. 2 of the manuscript for details). The preliminary experiment presents two system histories obtained with the two heterogeneity models for given heterogeneity indexes. As with CF model, we observe using the SF model rapid formation of lanes with the static heterogeneity type M_1, while band patterns emerge with the dynamic heterogeneity type M_2 (compare Fig. 2 in the manuscript to Fig. S1). The convergence to lane and band patterns is slower for larger systems (see the dotted curves obtained on a three times larger system with 135 agents on a 15×9 m rectangle).

Phase transitions occur in stationary states as the heterogeneity indexes increase. The dynamics range from disorder states to ordered states with lane or band patterns and polarised order parameters, see Fig. S2. This holds for heterogeneity relying on agent speed features (heterogeneity index δ_s, see Fig. S2) or on the agent size (heterogeneity index δ_l, see Fig. S3). However, the transition to lane pattern is specially laborious when dealing with the size parameter with SF model (see Fig. S3, top left panel). Indeed, the size parameter ℓ with SF model does not describe a hard-core exclusion between the agents as CF model does. On the other hand. the phase transitions to band patterns (heterogeneity type M_2) occur for a lower heterogeneity feature again with SF model (compare Figs. 3 and 4 in the manuscript to Figs. S2 and S3).

The phase transitions to lane and band patterns can be observed during the first minutes of simulations (see Fig. S4). However, in contrast to the results obtained with CF model, the system is not completely stabilised after 600 s, especially for the heterogeneity model M_1 (compare Fig. 5 in the manuscript to Fig. S4). SF model, being an inertial model of the second order, requires longer simulation times to describe stationary performances. Similarly to CF model, noising the dynamic clearly perturbs the lane formation (freezing-by-heating-effect, compare Fig. 6 in the manuscript to Fig. S5, left panels). While, oppositely, the band formation is robust against the noise (see Fig. S5, right panel).

Definition of the social force model In the social force model, the dynamics of an agent n with position $\mathbf{x}_n$ and neighborhood $\mathbf{X}_n$ is given by the second order differential equation

$$\ddot{\mathbf{x}}_n = F^{\text{SF}}_{\mathbf{p}_j}(\mathbf{X}_n) = \frac{1}{\tau}(V_j\,\mathbf{e}_0 - \dot{\mathbf{x}}_n) + \sum_{m \neq n} \varphi(\mathbf{e}_{mn}) \nabla U_j(\|\mathbf{x}_n - \mathbf{x}_m\|) + \sigma \xi_n. \tag{S1}$$

Here $V_j \geq 0$ is the desired speed, $\ell_j \geq 0$ the agent size, $\tau = 0.5$ s a relaxation time, while $\varphi(\mathbf{e}) = 1 - \cos(\pi - \hat{\mathbf{e}})$ is the vision field factor with $\mathbf{e}_{mn}$ the direction from m to n. As for CF model, $\mathbf{e}_0 = 0$ is the desired direction (polarity) and $U(x) = A_j \exp\big((\ell_j - x)/B\big)$ with parameters A_j and $B = 0.2$ m is a repulsive potential with the neighbors. A bi-dimensional white noise ξ_n (i.e. the time derivatives of two independent Wiener processes) with amplitude $\sigma > 0$ is used in Fig. S5. The model is simulated using an explicit Euler numerical scheme in deterministic cases, and using an Euler-Maruyama scheme for the simulation including a stochastic noise. The time step is $\delta t = 0.01$ s in both cases.

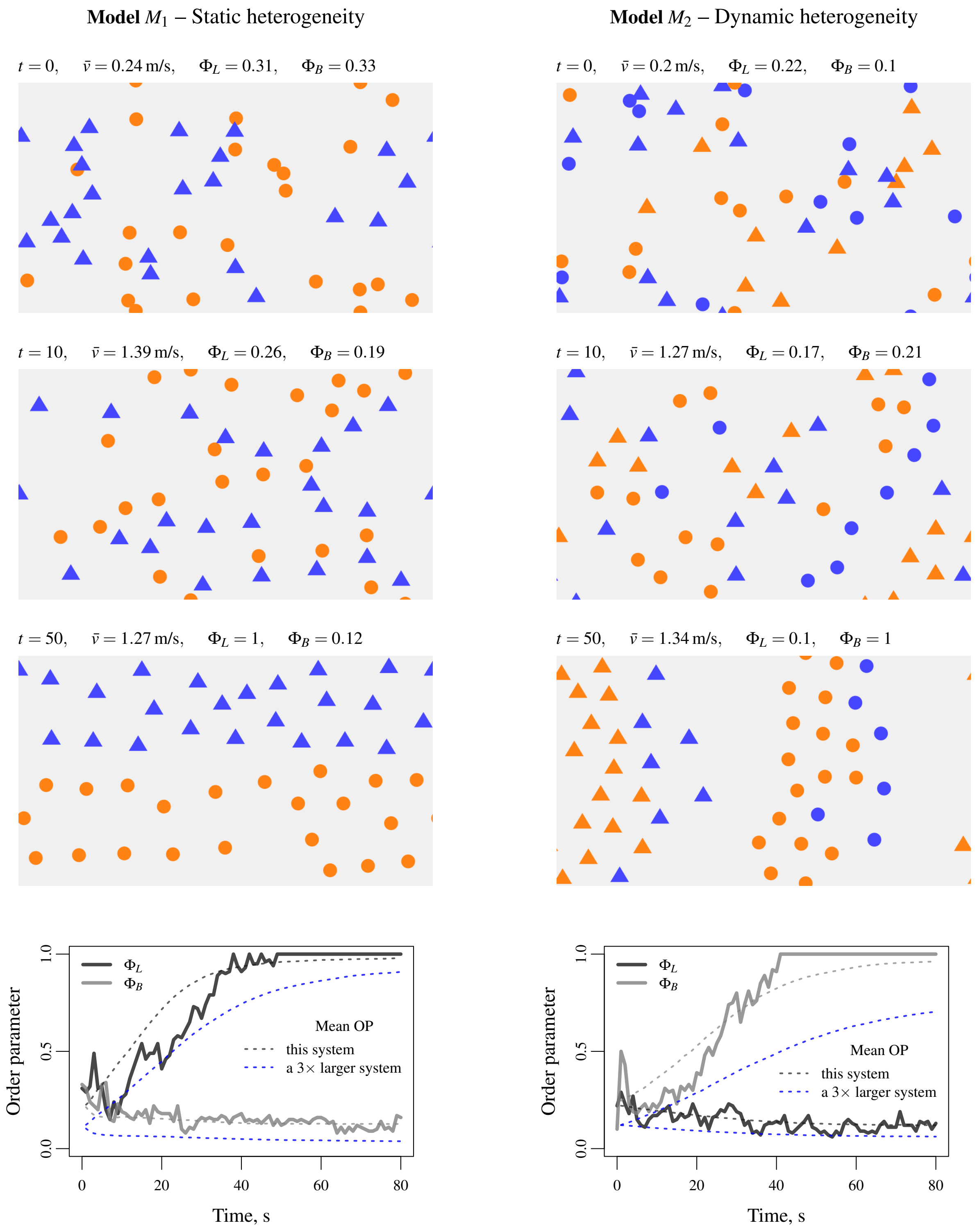

Figure S1. Typical histories for the model M_1 with heterogeneity in the agent characteristics for which lanes emerge: the lane order parameter Φ_L tends to one while the band order parameter Φ_B is close to zero (left panels, $\delta_s = 18$), and for the model M_2 with heterogeneity in the interactions where bands emerge, Φ_L is close to zero while Φ_B tends to one (right panels, $\delta_s = 10$). SF motion model, flow direction from left to right, periodic boundary conditions, random initial conditions.

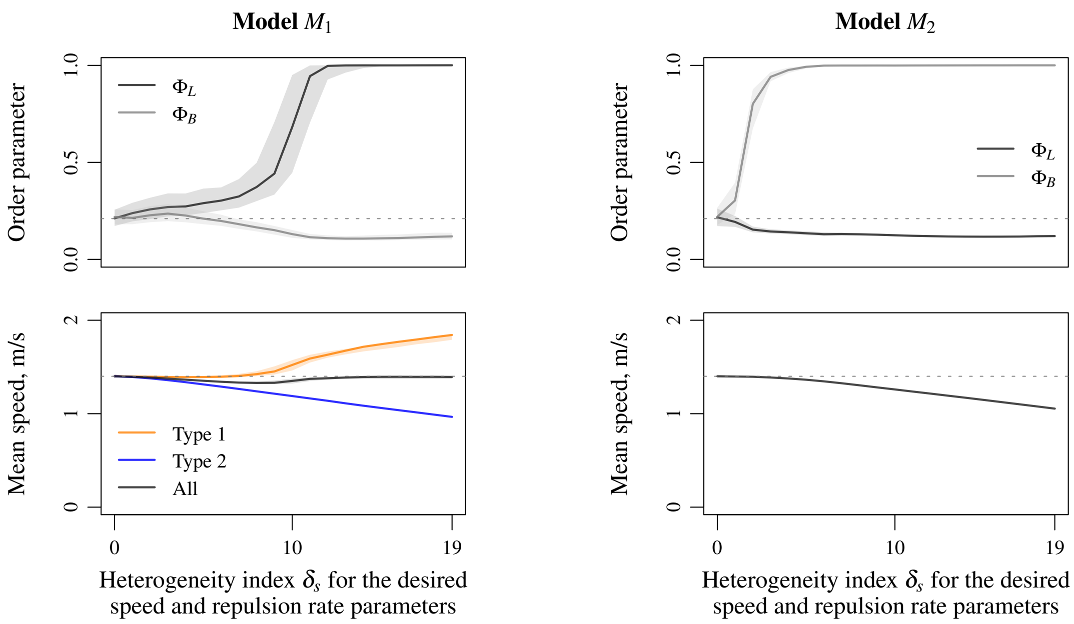

Figure S2. Lane and band order parameters (top panels) and mean speed (bottom panels) according to the heterogeneity index of agent speed features for the static heterogeneity model M_1 (left panels) and for the dynamic heterogeneity model M_2 (right panels) with the SF model. We observe qualitatively similar phase transitions as those obtained with the CF model, compare with Fig. 3 in the manuscript.

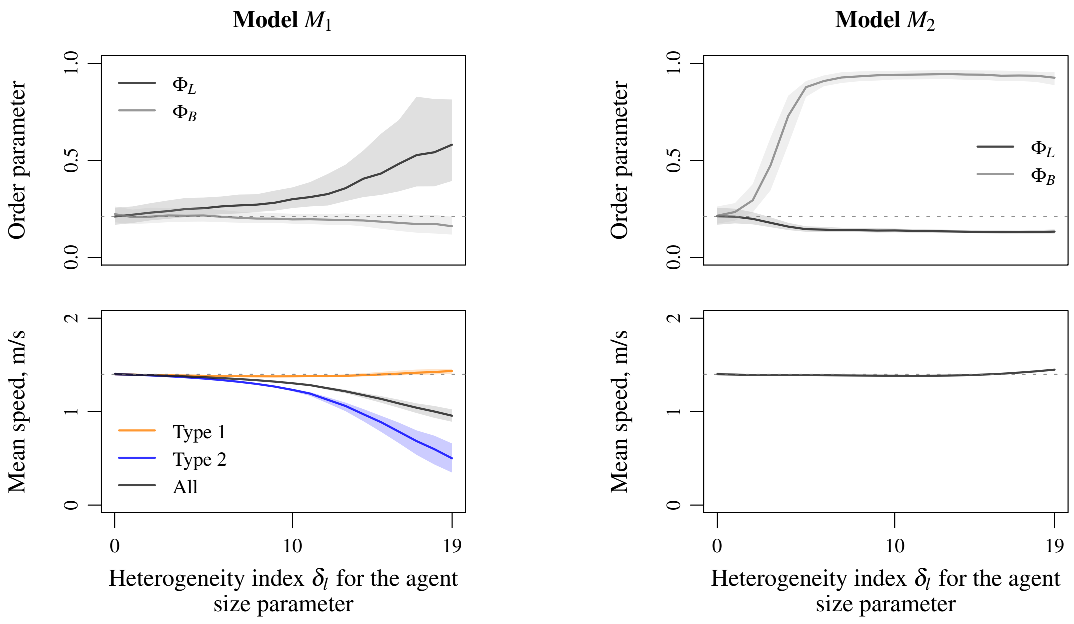

Figure S3. Lane and band order parameters (top panels) and mean speed (bottom panels) according to the heterogeneity index of agent size for the SF model. In contrast to CF model (see Fig. 4 in the manuscript), the transition to lane patterns is slower in absence of strict exclusion rules between the agents.

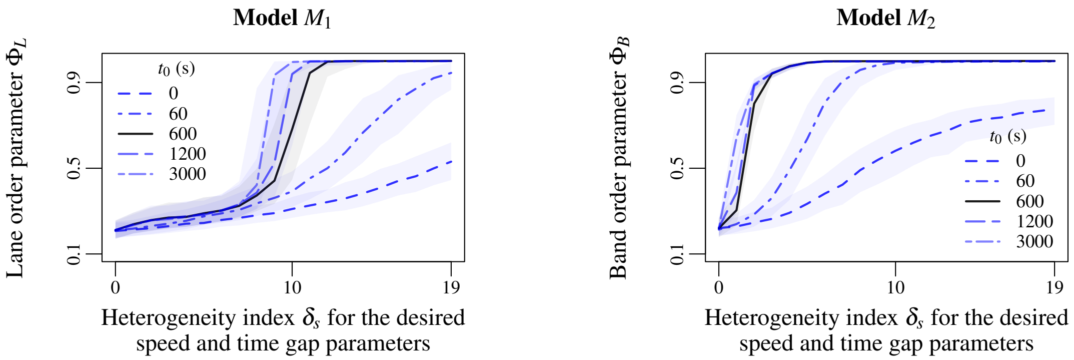

Figure S4. Lane order parameter for the static heterogeneity model M_1 (left panel), and band order parameter for the dynamic heterogeneity model M_2 (right panel) according to the heterogeneity index δ_s of agent speed features. The different curves correspond to different simulation times $t_0 = 0$, 60, 600, 1200 and 3000 s before starting the measurements (random initial conditions). As for the CF model, the phase transition to lane and band patterns relatively fast emerges with the SF model. It can be observed during the first minutes of simulation. However, the system is not completely stabilised after 600 s.

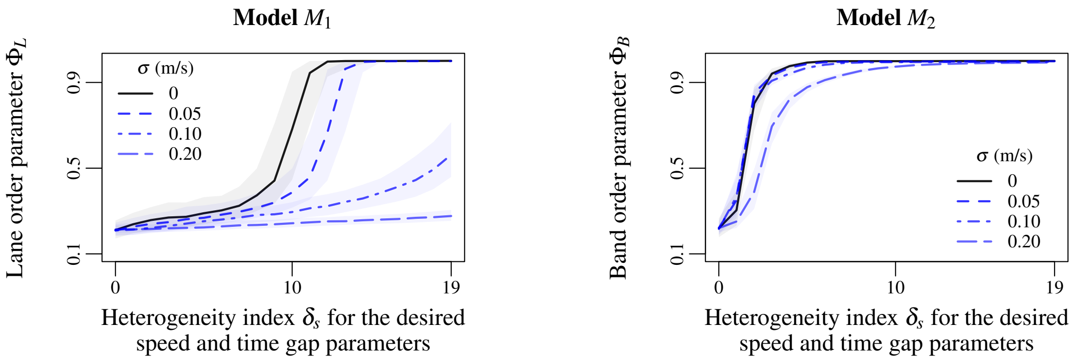

Figure S5. Lane order parameter for the static heterogeneity model M_1 (left panel), and band order parameter for the dynamic heterogeneity model M_2 (right panel) according to the heterogeneity index δ_s of agent speed features. The different curves correspond to different noise amplitudes $\sigma = 0$, 0.1, 0.2 and 0.5 m/s in the dynamics. The noise clearly perturbs the lane formation (left panel, freezing-by-heating-effect). Oppositely, the band formation is robust against the noise (right panel).

Setting of the parameters The default values for the parameters $\mathbf{p} = (\ell, V, A)$ of the SF model are based on the setting proposed in the literature[1]: $\ell = 0.3$ m, $V = 1.5$ m/s, and $A = 3$ m/s^2. Starting from the default values, we vary using heterogeneity indexes the parameter settings $\mathbf{p}_1 = (\ell_1, V_1, A_1)$ and $\mathbf{p}_2 = (\ell_2, V_2, A_2)$.

- In the analysis of the speed heterogeneity (heterogeneity level δ_s, cf. Figs. S1, S2, S4 and S5) the expulsion rate A ranges into $[2,4]$ m/s^2 by step of 0.05 m/s^2: $A_1 = A - 0.05\delta_s$, $A_2 = A + 0.05\delta_s$, $\delta_s = 0, \ldots, 19$. The desired (maximal) speed V, as with CF model, ranges into $[1,2]$ m/s by step of 0.025 m/s: $V_1 = V - 0.025\delta_s$, $V_2 = V + 0.025\delta_s$, $\delta_s = 0, \ldots, 19$. Note that $\tau_j = V_j / A_j$ systematically holds for all $j = 1, 2$. The parameter $\ell_1 = \ell_2 = \ell$ for the agent size remains constant.
- In the analysis of the size heterogeneity (heterogeneity level δ_l, cf. Fig. S3) the parameter ℓ, as with CF model, ranges into $[0, 0.9]$ m by step of 0.015 m decreasing and 0.03 m increasing: $\ell_1 = \ell - 0.015\delta_l$, $\ell_2 = \ell + 0.03\delta_l$, $\delta_l = 0, \ldots, 19$. The remaining parameters for the agent speed $V_1 = V_2 = V$, and $A_1 = A_2 = A$ are constant.

References

1. Helbing, D. & Molnár, P. Social force model for pedestrian dynamics. *Phys. Rev. E* **51**, 4282–4286 (1995).
